# A triphosphate tunnel metalloenzyme from pear (PbrTTM1) moonlights as an adenylate cyclase

**DOI:** 10.3389/fpls.2023.1183931

**Published:** 2023-06-22

**Authors:** Ye Yuan, Yuye Liu, Shuangjiang Chen, Lili Wang, Lixin Wang, Yahong Niu, Xin Zhao, Zhihui Zhao, Zhiguo Liu, Mengjun Liu

**Affiliations:** ^1^ College of Horticulture, Hebei Agricultural University, Hebei, China; ^2^ Research Center of Chinese Jujube, Hebei Agricultural University, Hebei, China

**Keywords:** pear, adenylyl cyclase, triphosphate tunnel metalloenzyme, moonlighting protein, tertiary structure

## Abstract

Adenylyl cyclase (AC) is the vital enzyme for generating 3′,5′-cyclic adenosine monophosphate, an important signaling molecule with profound nutritional and medicinal values. However, merely, a dozen of AC proteins have been reported in plants so far. Here, a protein annotated as triphosphate tunnel metalloenzyme (PbrTTM1) in pear, the important worldwide fruit plant, was firstly identified to possess AC activity with both *in vivo* and *in vitro* methods. It exhibited a relatively low AC activity but was capable of complementing AC functional deficiencies in the *E. coli* SP850 strain. Its protein conformation and potential catalytic mechanism were analyzed by means of biocomputing. The active site of PbrTTM1 is a closed tunnel constructed by nine antiparallel β-folds surrounded with seven helices. Inside the tunnel, the charged residues were possibly involved in the catalytic process by coordinating with divalent cation and ligand. The hydrolysis activity of PbrTTM1 was tested as well. Compared to the much higher capacity of hydrolyzing, the AC activity of PbrTTM1 tends to be a moonlight function. Through a comparison of protein structures in various plant TTMs, it is reasonable to speculate that many plant TTMs might possess AC activity as a form of moonlighting enzyme function.

## Introduction

The 3′,5′-cyclic adenosine monophosphate (cAMP) was firstly found as a heat-stable factor mediating the action of epinephrine and glucagon on activations of liver phosphorylase in 1956 (Sutherland and Rall, 1957; Sutherland, 1972). The subsequent research studies demonstrate that cAMP is an important second messenger and mediates various physiological and biochemical processes, including metabolism, transcription, and cell growth, mainly by activating protein kinase A (Walsh et al., 1968; Taskén et al., 1997). cAMP also exhibits intriguing nutritional and medical values, such as participating in converting short-term to long-term memory (Goelet et al., 1986; Alberini et al., 1995) and displaying diverse roles in regulating tumors (Zhang et al., 2020). Whereas in plants, even the existence of cAMP seemed to be debatable for some time encountered by the much lower level of cAMP compared to animals (Assmann, 1995). Hitherto, the reports on the cAMP content in fruits are still limited. Liu and Wang (1991) reported that the cAMP content in the worldwide fruit pear (*Pyrus* × *bretschneideri*) was under 15.0 pmol/g·Fw.

Meanwhile, the low level of cAMP also hindered the research studies on its synthesis enzyme, adenylyl cyclase (AC), in plants. It is widely known that ACs from prokaryotes to eukaryotes are highly polymorphic and could be classified into six classes, I–VI ACs, by their structural features (Khannpnavar et al., 2020). Among them, the class III ACs are sub-categorized into nine types, eight transmembrane ACs and one soluble AC (Linder and Schultz, 2003). In contrast, only 23 plant enzymes from seven species have been reported to exhibit AC activity so far, and most of them were obtained by searching the AC catalytic center motif that composed of 14 amino acids (Wong and Gehring, 2013; Gehring and Turek, 2017), like *Arabidopsis thaliana* clathrin assembly protein (AtClAP), *Arabidopsis thaliana* K^+^-uptake permease 5 (AtKUP5) (Al-Younis et al., 2018), *Arabidopsis thaliana* K^+^-uptake permease 7 (AtKUP7) (Al-Younis et al., 2015), *Arabidopsis thaliana* leucine-rich repeat protein (AtLRRAC1) (Ruzvidzo et al., 2013; Bianchet et al., 2019), *Arabidopsis thaliana* pentatricopeptide repeat protein (AtPPR) (Ruzvidzo et al., 2019), *Arabidopsis thaliana* 9-cis-epoxycarotenoid dioxygenase 3 (AtNCED3) (Al-Younis et al., 2021), *Arabidopsis thaliana* AUXIN-SIGNALING F-BOX 5 (AtAFB5), *Arabidopsis thaliana* AUXIN-SIGNALING F-BOX 1 (AtAFB1) and *Arabidopsis thaliana* TRANSPORT INHIBITOR RESPONSE 1 (AtTIR1) (Qi et al., 2022).

It should be noted that most of the plant ACs come into existences disguised as other proteins (Moutinho et al., 2001), especially those in large gene families that were screened out by searching putative AC core motifs. Nevertheless, there are exceptions, for instance, protein that Combined AC with PDE in *Marchantia* polymorpha (MpCAPE)-AC from basal plant *Marchantia polymorpha* exhibiting significant similarity with ACs of class III (Kasahara et al., 2016), whereas *Hippeastrum hybridum* adenylyl cyclase protein (HpAC1) (Świeżawska et al., 2014), *Brachypodium distachyon* triphosphate tunnel metalloenzyme 3 (BdTTM3) (Świeżawska et al., 2020), *Malus domestica* triphosphate tunnel metalloenzyme 1 (MdTTM1), and *Malus domestica* triphosphate tunnel metalloenzyme 2 (MdTTM2) (Yuan et al., 2022) consisting of only about 200 amino acids. Despite this, the experimentally confirmed plant ACs still show restricted conservation and multi-functionality that corresponds to their wide distribution in plants, including basal, herbaceous, and woody plants, and participation in various biological processes. In addition, the AC activities of these plant proteins are extremely limited. Thus, the plant proteins verified with AC activity are rarely designated as AC, on the contrary, retaining their original names. Moreover, because cAMP plays diverse roles in plants like influencing pollen tube growth and reorientation (Tsuruhara et al., 2001), modulating ion flux (Zelman et al., 2012; Lu et al., 2016; Wang et al., 2020), regulating cell division (Ehsan et al., 1998; Sabetta et al., 2016), conferring biotic and abiotic stress tolerance (Ordoñez et al., 2014; Sabetta et al., 2019), and mediating auxin-induced transcriptional regulation (Blanco et al., 2020; Qi et al., 2022), it is speculated that plant cells contain a considerable number of AC to fulfill these diverse functions. However, none of the genes in the pear genome are annotated as *ACs*, and none of the proteins with AC activity have been identified in pear so far.

The CYTH superfamily is designated by combining CyaB, the class IV AC, and thiamine triphosphatase. It was reported that the orthologies of CYTH superfamily could be widely found in bacteria, archaeon, plants, and animals, but not in fungi until Cet1 RNA triphosphatase from *Saccharomyces cerevisiae* was reported to display extremely similar structure, the topologically closed tunnel, with bacterial and archaeal CYTH proteins. In view of the fact that huge differentiation of amino acid sequences between Cet1 RNA triphosphatase with other members of CYTH, a larger superfamily, triphosphate tunnel metalloenzymes (TTMs), was proposed. It is known that TTM is a conservative superfamily widely distributed in archaeons, bacteria, fungi, animals, and plants (Bettendorff and Wins, 2013). In the previous studies, the class IV ACs, found in bacteria *Aeromonas hydrophila* (Sismeiro et al., 1998) and *Yersinia pestis* (Smith et al., 2006), were classified into the branch of TTM. Considering that no *AC* genes were annotated in the genome of the worldwide distributed fruit tree pear, whereas a few plant TTMs have been experimentally confirmed to produce AC activities, including MdTTM1 and MdTTM2 from fruit tree apple (Yuan et al., 2022), ergo, we focused on detecting the AC activity of the TTMs in pear and trying to uncover its AC catalytic characteristics.

## Materials and methods

### Sequence analysis of candidate proteins

The sequence information of candidate AC proteins in pear (PbrTTMs) and other horticultural plants was obtained by aligning with the sequence of BdTTM3 with protein Basic Local Alignment Search Tool, or BLASTp, in National Center for Biotechnology Information (NCBI; https://www.ncbi.nlm.nih.gov/) (Johnson et al., 2008). Multiple sequence alignment of candidate amino acid sequences was alignment with software DNAMAN (version 6.0) (Wang, 2015). The conserved motifs were discovered with Multiple Em Motif Elicitation suite (MEME; version 5.5.0; https://meme-suite.org/meme/tools/meme) (Bailey et al., 2015). The number of possible candidate motifs was set to 8.

### Overexpression and purification of PbrTTM1

The coding nucleotide sequence of PbrTTM1 was optimized to facilitate expression in prokaryotic cells. The recombinant bacterial expression plasmid, which has an optimized sequence linked to the pET-28a vector, was synthesized by General Biosystems (Anhui) Co., Ltd. The heat-shock method was used to transform the foreign recombinant plasmid into BL21(DE3)-competent *Escherichia coli*. The positive colonies were isolated from Luria-Broth (LB) agar that was spread plated with transformed competent cells and then inoculated in LB medium to overexpress target proteins that were induced with 1 mM isopropyl-1-thio-b-D-galactopyranoside (IPTG) at 20°C for 12 to 16 h. Rough extracted proteins were sonicated with ultrasonic generator homogenizer and purified with Ni-affinity chromatography according to the manufacturer’s protocol. sodium dodecyl sulfate–polyacrylamide gel electrophoresis (SDS-PAGE) was applied to review the quality of purification (Liu et al., 2023).

### Determination of catalytic property

Purified proteins were used to in the *in vitro* enzymatic experiments. The reaction system of 500-μl volume was applied where Tris-HCl (50 mM, pH 7.5), 2.5 mM divalent cation, 20 μg of purified protein, and diverse concentration of substrate were added. The reaction system was place under 95°C conditions to terminate the reaction. The AC catalytic activity was detected by measuring the cAMP level in reaction products with the Plant cAMP ELISA Kit (MM-6255101, Jiangsu Meimian Industrial Co., Ltd.). The content of free inorganic phosphate in the reaction product was used as the indicator of hydrolysis activity. Its quantification was accomplished with the Malachite Green Phosphate Assay Kits (POMG-25H, BioAssay Systems), and the absorbance at 630 nm was measured on a plate reader.

### *In vivo* verification of AC activity

*E. coli cyaA*-mutant SP850 strain [lam-, el4-, relA1, spoT1, cyaA1400 (:kan), thi-1] was used to verify the AC activity *in vivo* (Shah and Peterkofsky, 1991). The recombinant prokaryotic expression vector and empty vector, as negative control group, were separately transformed into SP850 strain with heat-shock method, and the strain harboring empty vector was the negative control group. The *E. coli* with normal functions was as the positive control group. The bacteria was cultured in LB media and induced with 0.5 mM IPTG (Beijing Solarbio Technology Co., Ltd.). When their concentration reached OD600 of 0.6, the bacteria were streaked on MacConkey agar to observe color changes (Moutinho et al., 2001).

### Structural analysis and molecular docking simulation

The tertiary structure models were downloaded from AlphaFold Protein Structure Database (https://alphafold.ebi.ac.uk/) (Varadi et al., 2022) for structural analysis. Whereas, the molecular model of ligand model was downloaded from Protein Data Bank (http://www.wwpdb.org/). Molecular docking simulation was performed using Autodock vina (version 1.2.0). The docking simulation was set to an exhaustiveness of 200. Conservation analysis was conducted on the ConSurf server (https://consurf.tau.ac.il/consurf_index.php) to perform and visualization of protein tertiary structure (Ashkenazy et al., 2016). Specifically, the method of automatically aligning the primary structure with UniRef90 database was used for conservation analysis (Suzek et al., 2015). The visualization work of tertiary structure model, conservation analysis, and molecular docking simulation was realized with PyMOL (version 2.5) (Seeliger and de Groot, 2010).

## Results

### Identification of candidate ACs from pear

The primary structure of BdTTM3 with AC activity was used to search homologous proteins from pear with BLASTP programs. The results showed that two orthologous genes were screened out from pear genome database, which were annotated as TTM as well. Hence, the two TTM genes were designated as *PbrTTM1* (LOC103928933) and *PbrTTM2* (LOC103953859) (**Supplementary Table 1**). These two genes showed a certain extent of similarity. The repetitive rate between *PbrTTM1* and *PbrTTM2* reached 54.73%. The alignment of primary structures also provided the conserved sequences in PbrTTMs, such as MEVEVKLRL that harbored the typical TTM motif EXEXK.

**Figure 1:**
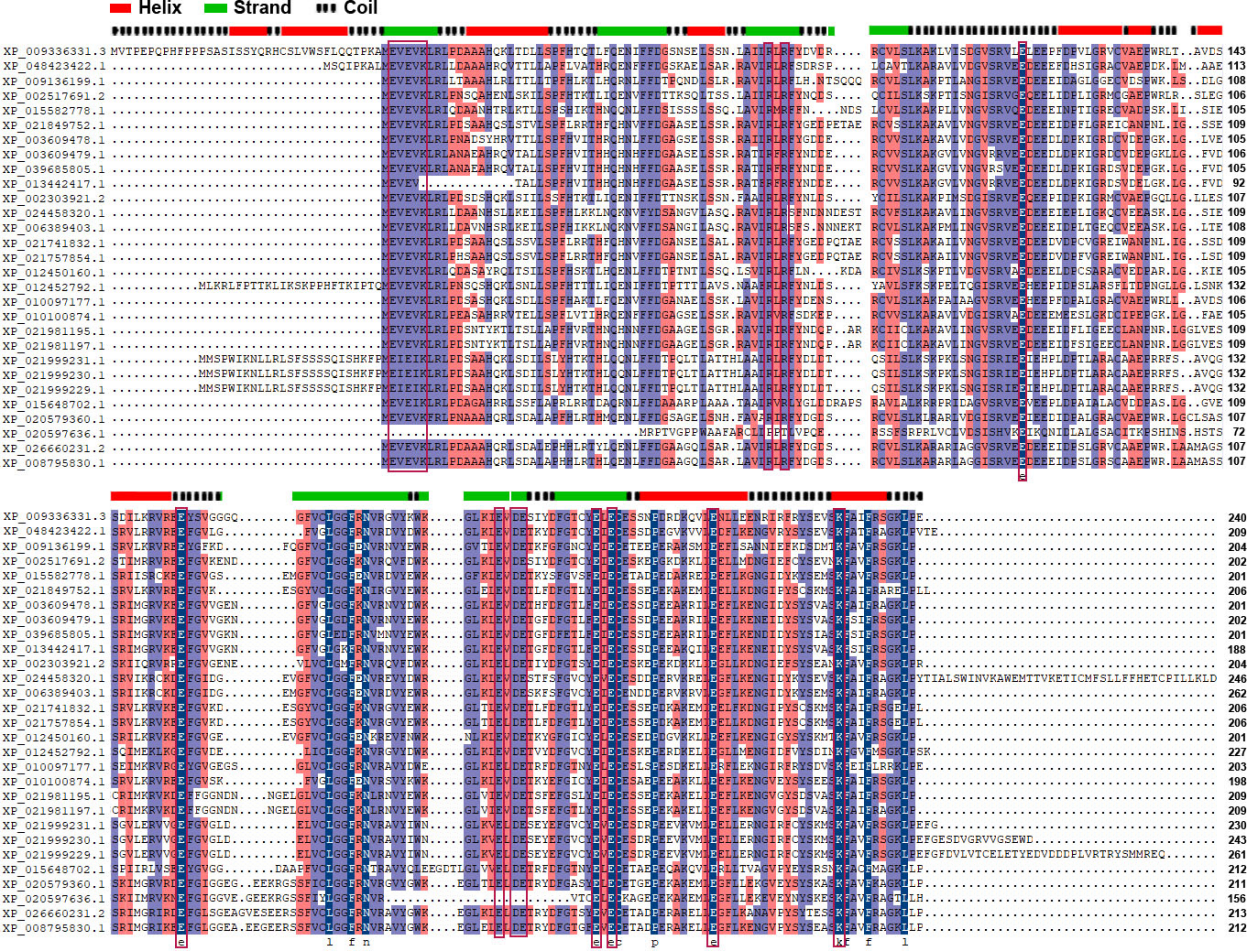
The alignment analysis of amino acid sequences of PbrTTMs and orthologous proteins. The EXEXK motif and conserved charged residues were highlighted with red lines. The predicted secondary structure of PbrTTM1 was on the top of alignment results corresponding to the sequence of PbrTTM1. The motif analysis of TTMs from 12 plant species.

We found that the conservation of TTMs collected from 12 plant species approaching 46.71% (**Figure 1**) and the typical motif EXEXK only absent in two proteins (**Supplementary Figure 1**). The regions with higher conservation in PbrTTMs were highlighted with light purple and dark blue color. The majority of them distributed on the β strands to construct the catalytic tunnel, referring to the position of protein secondary structure. From the result of discovery of conserved motifs, most TTMs contained six conserved motifs covering the vast majority of sequences. Among them, PbrTTM1 harbored all the six motifs, whereas motif 5 was missed in PbrTTM2. Therefore, the more conserved protein, PbrTTM1, was selected as the candidate AC for verification.

### Verification of AC activity

**Figure 2:**
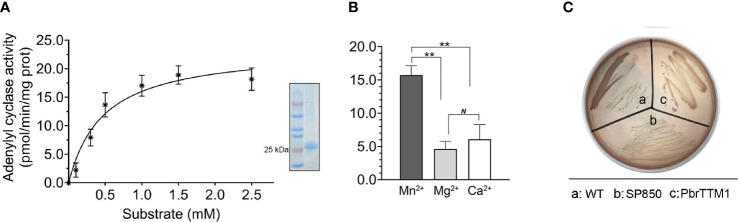
Verification of adenylyl cyclase activity of PbrTTM1. **(A)** The catalytic efficiency of PbrTTM1 in response to different concentrations of substrate ATP. The fitted curve, computed with GraphPad Prism (version 9.4.0), was 0.94. The SDS-PAGE analysis of purified protein was shown nearby. **(B)** Catalytic rate of PbrTTM1 assisted with different metal ion including Mn^2+^, Mg^2+^, and Ca^2+^. The values are the mean of three independent replicates. Error bars represent standard deviation (**P< 0.01; *N*, not significant). **(C)** *In vitro* verification test with AC-deficient *E coli cyaA* mutation. Colonies of host cells transformed with a recombinant vector are displayed the same color with WT, whereas cells harboring an empty vector were white on MacConkey agar.

To detect the AC activity of PbrTTM1, the *in vitro* enzymatic experiments were performed. The protein for trial was obtained by purifying the crude protein product from prokaryotic expression system. The results demonstrated that PbrTTM1 was capable of performing AC activity, however, with limited efficiency. Corresponding parameters of the enzyme kinetics were Vm = 20.7 pmol/min/mg protein, Km = 0.31 (**Figure 2A**). The restricted AC catalytic rate accorded with the prevailing efficiency of experimentally verified plant ACs whose catalytic efficiencies were all under 1.0 nmol/min/mg protein. To be more specific, the AC activity of PbrTTM1 is relatively similar to that of AtNCED3, NbAC, AtPPR, AtKUP5, AtTIR1, etc., ranging from 1.0 to 20.0 pmol/min/mg protein, which could be classified as relatively low level. As a metalloenzyme, PbrTTM1 displayed varied AC activity with different divalent cations added in the reaction system. Apparently, Mn^2+^ gave the most effective support for AC activity of PbrTTM1 comparing to Ca^2+^ and Mg^2+^ (**Figure 2B**), which was the suitable divalent to class III ACs.

*E. coli* strain SP850 is a suitable host to qualitatively validate the AC activity of protein, for the AC deficiency of itself. Therefore, in the additional experimental confirmation, this *E. coli* strain was applied to test the AC activity of PbrTTM1 *in vivo* by observing the color of colonies. The result is distinct that the colonies of test group, heterologously transformed PbrTTM1 SP850, shared the same color with normal *E. coli* strain on MacConkey agar medium (**Figure 2C**), whereas the negative control colonies were oyster white, which suggested that PbrTTM1 could successfully rescue the AC deficiency of SP850.

### Structural analysis of PbrTTM1

Hitherto, many of verified plant ACs possess the core AC motif ([RKS]X[DE]X(9,11) [KR]X(1,3)[DE]) that only harbors a small number of conserved functional amino acid residues conferring substrate specificity, ATP binding, and divalent cation binding. However, the core AC motif was not found in the amino acid sequence of PbrTTM1. Then, we predicted the tertiary structure of PbrTTM1 by AlphaFold 2.0 to further speculate the mechanism of PbrTTM1 possessing AC activity.

**Figure 3:**
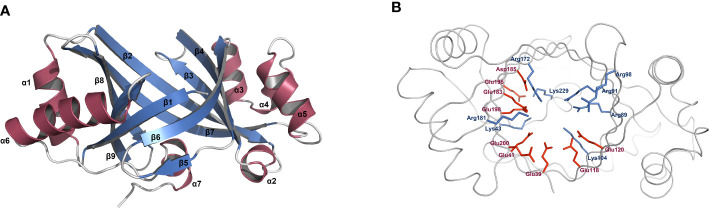
Structural analysis of PbrTTM1. **(A)** Overview of PbrTTM1 fold. **(B)** Distribution of basic and acidic residues the inside the catalytic site. Basic and acid residues were presented with blue and red sticks, respectively. The model of PbrTTM1 was constructed with AlphaFold 2.0, and images were created with PyMOL (version 2.5).

From the overall view of its tertiary structure model, PbrTTM1 basically abode by the typical structural characteristics of TTM (**Figure 3A**). The monomer is roughly egg-shaped with the dimensions of 35.8 × 38.6 × 56.7 Å, similar with the structure of AtTTM3 from *Arabidopsis thaliana* and YpAC from *Y. pestis*. PbrTTM1 contains nine β strands, and eight of them form the barrel cavity with the order of β19872346. There are seven helices asymmetrically distributed on the two sides and bottom of the barrel cavity. Specifically, α1 and α6 are on the side nearby β9; α3, α4, α5, and α2 are nearby β4; α7 locates on the side nearby C-terminal of the protein. Inside the β-stranded barrel cavity, basic and acidic residues occupy a large percentage. As shown in **Figure 3B**, the side chains of eight basic residues and nine acidic residues that point to the tunnel cavity are shown with blue and red sticks, and some of them probably function in cAMP synthesis. On the contrary, the hydrophobic residues are barely distributed inside the tunnel but surround the catalytic pocket (**Supplementary Figure 2**).

**Figure 4:**
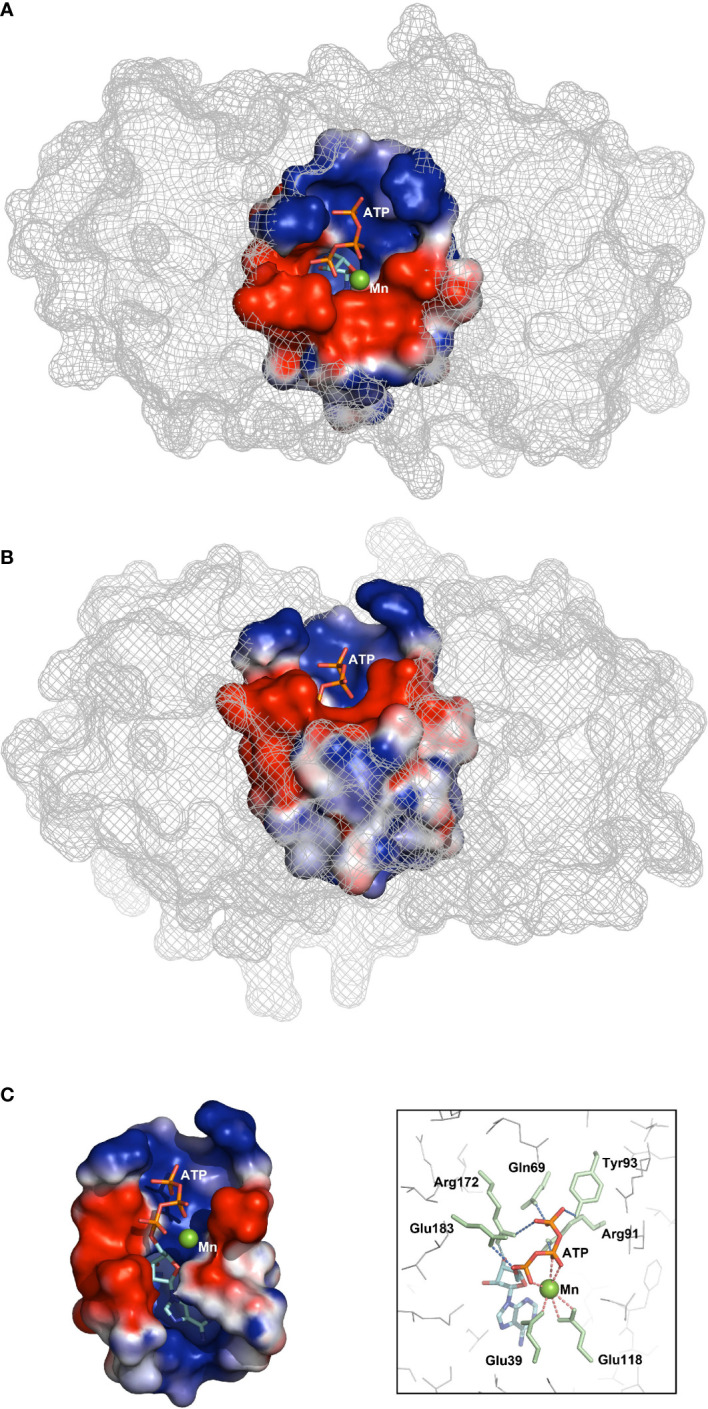
Docking simulations of ligand ATP to PbrTTM1. Vertical **(A)**, front **(B)**, and interior (**C**, left) views of the docking result of ligand to PbrTTM1; free energy of −6.9 kcal/mol. The box shows potential residues involved in catalytic process.

To speculate the potential mechanism of AC activity under the circumstance of being short of core AC motif, semi-flexible docking simulation was conducted with the help of Autodock vina (version 1.2.0). Among the docking results, the one with lowest free energy (−6.9 kcal/mol) was selected as the final outcome (**Figure 4**), which, intriguingly, presented a rational ligand pose simultaneously. The potential interaction between the residues and the ligand in the outcome could suggest a mechanism that is similar to the bimetallic ion catalysis mechanism. It could be seen that the ribose and adenosine moiety locates inside the catalytic pocket approaching to Lys229, whereas the triphosphate group extends to the portal of the pocket. The residues around triphosphate group, involving Arg91, Arg172, Glu183, Gln69, and Tyr93, are capable of H-bonding to phosphate oxygen, which possibly stabilizes the triphosphate part. Three phosphate oxygen atoms of Pα and Pβ coordinate with Mn^2+^, the proper divalent cation for AC activity. In addition, Mn^2+^ also coordinates two acidic residues Glu39 and Glu118 in the catalytic tunnel, which results in an octahedral structure constituted with atoms coordinating with divalent cation. Coordination with Mn^2+^ could enhance the electropositivity of Pα, thereby promoting nucleophilic attack by the O3′ atom. The deprotonation of O3′, which was thought to initiate cyclization, was speculated to be accomplished by the nearby basic residue Arg172. Deprotonation could also be accomplished by reducing the pKa of O3′ through introducing the second divalent cation, which might be hindered by the crowded catalytic tunnel pocket.

**Figure 5:**
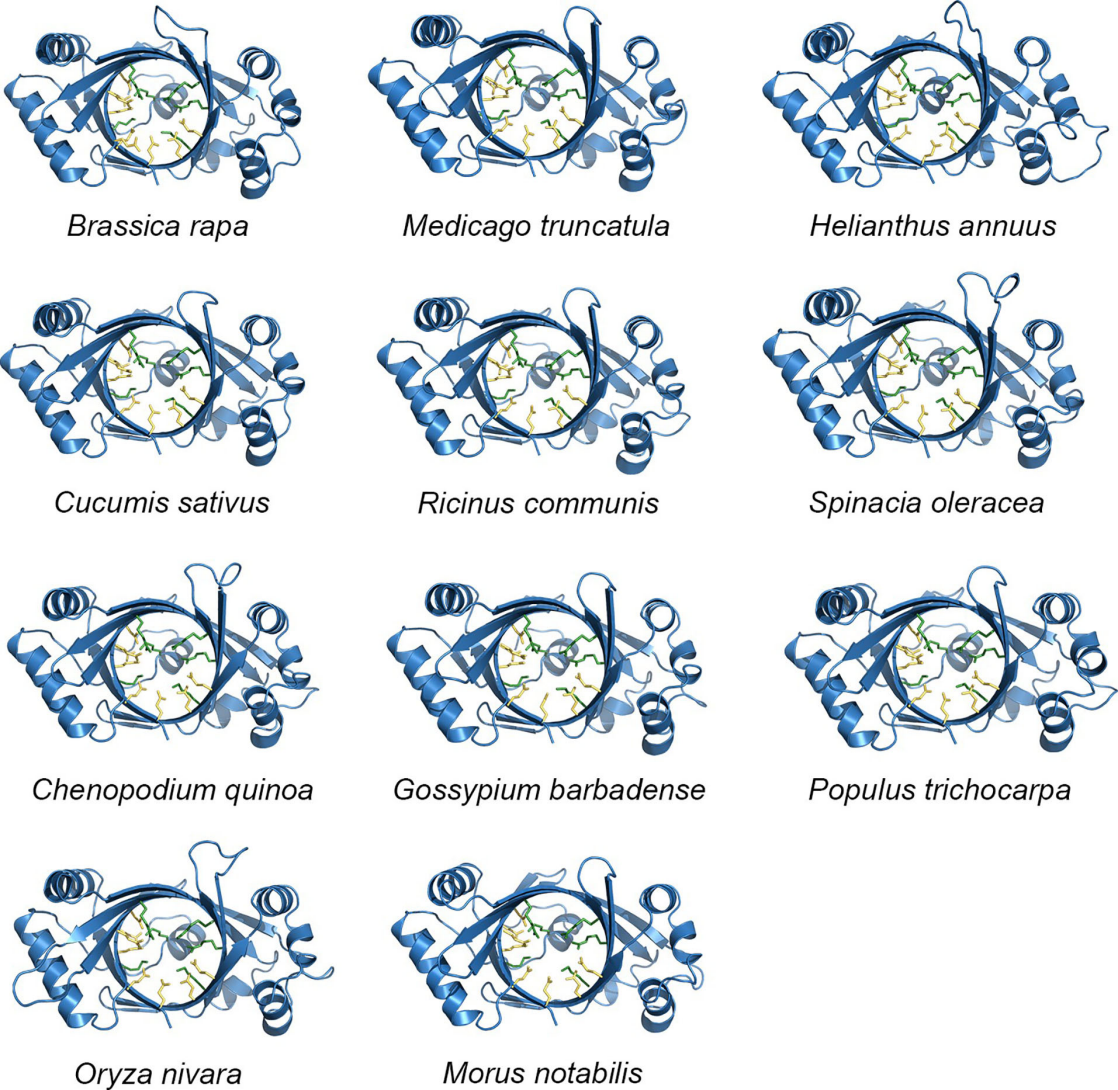
An overview of senior structure sketches of TTM proteins from other plants. The yellow and green sticks represent acidic and basic residues inside the tunnel, respectively. The conservation on tertiary structure supported by the overall structures and the marked residues may indicate functional consistency.

**Figure 6:**
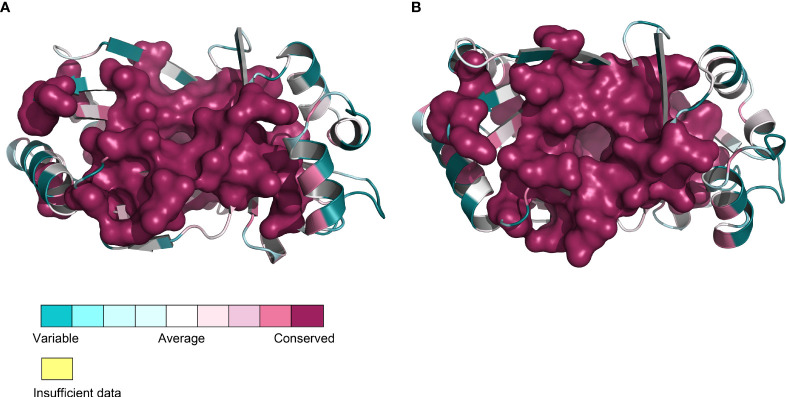
Front **(A)** and vertical **(B)** views of PbrTTM1 colored according to the conservation. The most conserved part was deep red and highlighted with surface model.

As previously noted, the amino acid sequence identity among TTM proteins from 12 different plant species was found to be 46.71%, with most sequences exhibiting the presence of six common motif regions. The TTMs were conserved, to some extent, during evolution according to the similarity levels of the amino acid sequences and the conserved motifs, and the similarity might extend to the tertiary structure as well. Subsequently, we utilized predictive analysis techniques to investigate the tertiary structures of TTM proteins from these plant species. The results indicate that, irrespective of diverse protein sources, the total number and distribution pattern of secondary structures within the protein structures remained highly conserved, except for the coils exhibiting a certain variation that, of course, might be attributed to the computational algorithms of the prediction software. Furthermore, the quantity and location of charged residues inside tunnels were found to be conserved, including the crucial functional residues predicted within PbrTTM1 (**Figure 5**). Meanwhile, the conservation of PbrTTM1 produced with ConSurf server also showed that the most conserved region concentrated on the catalytic cavity and the variable positions mostly present on periphery of the helices that possibly maintain the stability of protein, which indicated functional consistency (**Figure 6**). Therefore, it was possible to hypothesize even other plant TTMs possessed potential weak AC function, considering extremely limited cAMP level in plant cells.

### The primary catalytic activity of PbrTTM1

**Figure 7:**
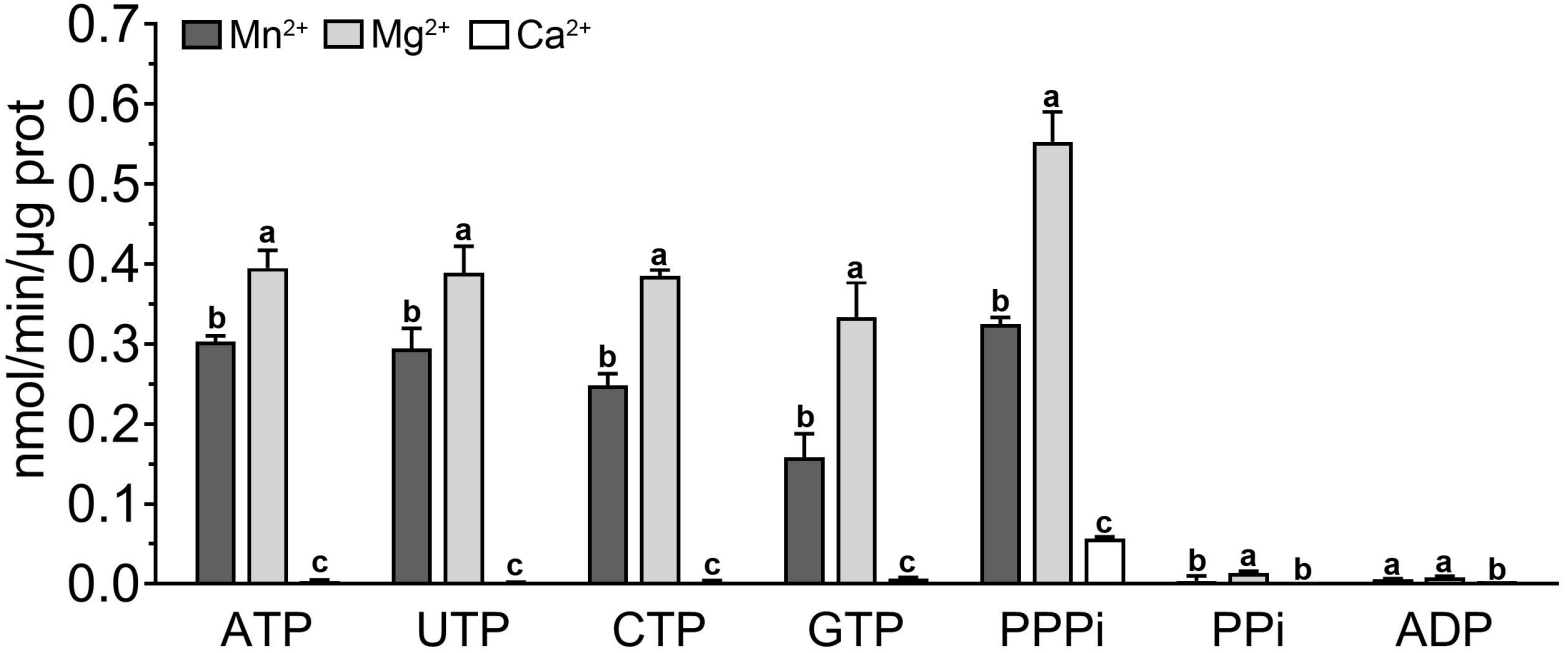
Catalytic efficiency of phosphate hydrolysis of PbrTTM1. The catalytic rates were collected by measuring the level of Pi generated in reaction system. Malachite Green Phosphate Assay Kits (POMG-25H) were used for determination. Three independent replications were performed. Error bars represent standard deviation.

Although the *in vivo* and *in vitro* experiments have confirmed the AC activity of PbrTTM1, its extremely restricted catalytic efficiency could not be ignored. PbrTTM1 might be a “moonlighting protein” that possesses primary function other than AC. The TTM protein super family harbors one category of AC, the class IV ACs found in bacteria whose catalytic efficiency are evidently exceeding the catalytic capacity of all the reported plant ACs. However, other proteins in TTM super family uniformly presented hydrolysis activity for substrates involving thiamine triphosphate, 5′-phosphopolynucleotide, nucleoside triphosphate, and inorganic triphosphate (Lima et al., 1999; Lakaye et al., 2002; Moeder et al., 2013). Thus, the hydrolysis activity of PbrTTM1 was determined as well. The results of *in vitro* enzymatic activity tests showed that PbrTTM1 possessed higher affinity to tripolyphosphate. Mg^2+^ and Mn^2+^ became the suitable divalent cations to support hydrolysis activity, especially Mg^2+^. PbrTTM1’s activity of hydrolyzing nucleoside triphosphate was lower than that of PPPase, like the ATPase activity achieving 0.39 nmol/min/μg protein, but it was still much higher than the efficiency of its AC activity (**Figure 7**). The efficient hydrolyzing capacity did not extend to substrate of pyrophosphate as well. Together, PbrTTM1 tends to be a moonlighting protein that exhibited limited AC activity and much stronger hydrolyzing ability for phosphate substrates. Moreover, it is not unreasonable to speculate that the hydrolysis activity for inorganic triphosphate and nucleoside triphosphate were the primary enzymatic activity for other plant TTM proteins.

## Discussion

BdTTM3 and HpAC1, homologous proteins from *Brachypodium distachyon* and *Hippeastrum*×*hybridum*, could occasionally convert ATP to cyclic AMP and pyrophosphate while exhibiting efficient catalytic activity of the nucleoside triphosphate and tripolyphosphate hydrolase, which were parallel with the catalytic property of PbrTTM1. Researchers also described the biological roles of these two proteins. The expression level of *BdTTM3* was significantly upregulated in mechanically wounded leaves of *B. distachyon* (Świeżawska et al., 2020). Similarly, the mRNA level of *HpAC1* in *H. hybridum* tissue increased after mechanical injury and *Phoma narcissi* infection (Świeżawska et al., 2014). Given the conservation of primary structure and catalytic characteristics between PbrTTM1 and these two proteins, it could be speculated that the physiological function of PbrTTM1 was probably related to the responses to biotic and abiotic stresses.

Although the physiological functions of plant ACs were reported as well, it was still unclear whether the physiological roles of these “moonlighting” enzymes were endowed by the finite AC activity. Particularly noteworthy is the distinction between TTMs, which possess AC activity, and other plant enzymes displaying AC function (Gehring and Turek, 2017). The TTMs have always been involved in the metabolism of triphosphate and the catalytic property demonstrated their much higher activity as the tripolyphosphatase and nucleoside triphosphatase, which means that more phosphates would be produced along with the progress of cyclization. Hence, it would be of necessity to exclude the influence caused by free phosphate (Michigami et al., 2018). The recent report that proved that the AC function of plant enzymes could be involved in signal transduction prompted the genetic method for physiological research studies. Qi et al. (2022) found that the AtTIR1 possessed weak AC activity (the Vmax of 10.45 fmol/min/μg protein) similar with other plant ACs, and one C-terminal AC motif. By means of mutating the AC motif, researchers demonstrated that the AC activity of AtTIR1 was vital for auxin mediated root growth inhibition. In addition, the first experimentally identified plant AC, ZmPSiP, also referred the function of AC activity with pharmacological method (Moutinho et al., 2001). The researchers imitated the abnormal growth of pollen tube in *Agapanthus umbellatus* caused by downregulated expression of homologous gene of ZmPSiP by employing AC inhibitor while rescuing the abnormal phenotype of expression-restrained mutant by exogenous cAMP (Moutinho et al., 2001). Similar pharmacological identification trial was also applied to explore the function of *ACs* from *Ziziphus jujuba*, however, in *A. thaliana* plant (Liu et al., 2023). It is notable that the *ZjACs* were also annotated as *TTM* in the genome database of NCBI. The authors found that the AC function of these “*TTM*” in jujube tended to involve in biological progresses like circadian rhythm pathway and hormone signal transduction pathway regulating many of the growth stages like germination, root growth, and flowering. In addition, taking into consideration the involvement of HpAC1 and BdTTM3 in biotic and abiotic stress, it is plausible that the subtle AC activities exhibited by these proteins, when genetically upregulated, may give rise to localized increments in cAMP levels. Consequently, this cAMP modulation might be capable of regulating various aspects of plant physiology, like plant growth and stress responses. Moreover, TTMs from diverse plants have been identified to perform AC activity (Świeżawska et al., 2014; Świeżawska et al., 2020; Yuan et al., 2022; Liu et al., 2023). Hence, it might be a viable way to discover more ACs in other plant species by testing the proteins tagged with TTM. Nevertheless, not all the homologous enzymes could act as the role of AC and even the verified TTMs only displaying weak AC activity. For instance, no AC activity was detected in TTMs from *A. thaliana* (Moeder et al., 2013; Ung et al., 2014; Ung et al., 2017). The senior structure of protein might explain this phenomenon. Although, inside the catalytic pocket, potential functional charged residues exist to initiate and accomplish catalysis, the inappropriate substrate gesture may hinder the AC activity. As observed by Kleinboelting et al. (2020), α,β-methylene ATP GppCp (APC) and guanosine-5’-[(β,γ)-methyleno]triphosphate (GppCp), two substrate analogs, were twisted in the catalytic center. In addition, the TTMs with weak AC activity might be capable of providing slightly higher odds for suitable substrate binding.

For horticultural plants especially fruits, the nutritional quality is another valuable research priority. After the discovery of cAMP in 1956, researchers have reported its notable role in signal transduction as the second messenger and in regulating numerous physiological processes (Zhang et al., 2020). Moreover, its notable role in regulating pathological processes like carcinoma and cardiovascular declares the remarkable nutritional and medicinal value of cAMP. Whereas, the abundance of cAMP in fruit tree crops, including pear, is notably restricted, with a prominent presence observed solely in the fruits of Chinese jujube. Hence, elevating the cAMP level in edible tissue would agree with the breeding aim of improving the nutritive quality with the help of bioengineering. The conserved TTM protein super family ubiquitous in plants may assist to achieve this demand. Although AC is not the predominant function of them, they still present a certain affinity to nucleoside triphosphate and high conservative tertiary structure with YpAC. It may be possible to improve the AC activity of these proteins by means of slight alternation and applied to directive breeding further. According to Kleinboelting et al., the twisted coordination pose of analogs substrate in the tunnel caused a deficiency in AC activity. The docking pose of ATP in this research seemed to be more similar with the ligand gesture coordinating to YpAC. It is possible that the crowded catalytic site and coil moiety at N-terminal hinder the function of AC.

## Data availability statement

The data presented in the study are deposited in the DRYAD repository, accession link: https://datadryad.org/stash/share/wB80inib_fNXD8LfITVkj_oofALmgZhHhs26JP7Dx_w.

## Author contributions

YY, YL and SC equally contributed to the study. Conceptualization: ML, ZL and YY. Methodology: YY, SC, YL and YN. Writing—original draft preparation: YY. Writing—review and editing: ZL, ML and LLW. Visualization: YY, ZL and LLW. Supervision: ZL and ML. Funding acquisition: ML. All authors contributed to the article and approved the submitted version.
