# Supplementary material for: A triphosphate tunnel metalloenzyme from pear (PbrTTM1) moonlights as an adenylate cyclase
Source: Front Plant Sci. 2023 Jun 22;14:1183931. doi: 10.3389/fpls.2023.1183931 (PMC10324617; doi:10.3389/fpls.2023.1183931)
Supplement: Supplementary file 1 [file Presentation_1.pdf]

Supplementary Table 1. Information of PbrTTMs cDNA and protein

Gene name	Gene symbol	CDS (bp)	ORF (bp)	Size (aa)	MW (kDa)	PI	CYTH-like_AC_IV-like (aa)
PbrTTM1	LOC103928933	723	723	240	27.34	5.57	39-220
PbrTTM2	LOC103953859	630	630	209	23.63	5.32	10-184

Supplementary Table 2. Docking simulation parameters

**Parameter**	**PbrTTM1**
Receptor	PbrTTM1.pdbqt
Ligand	ATP_flex.pdbqt
Center_x	-0.255
Center_y	-0.058
Center_z	0.421
Size_x	16
Size_y	16
Size_z	16
Num_modes	20
Energy_range	8
Exhaustiveness	200

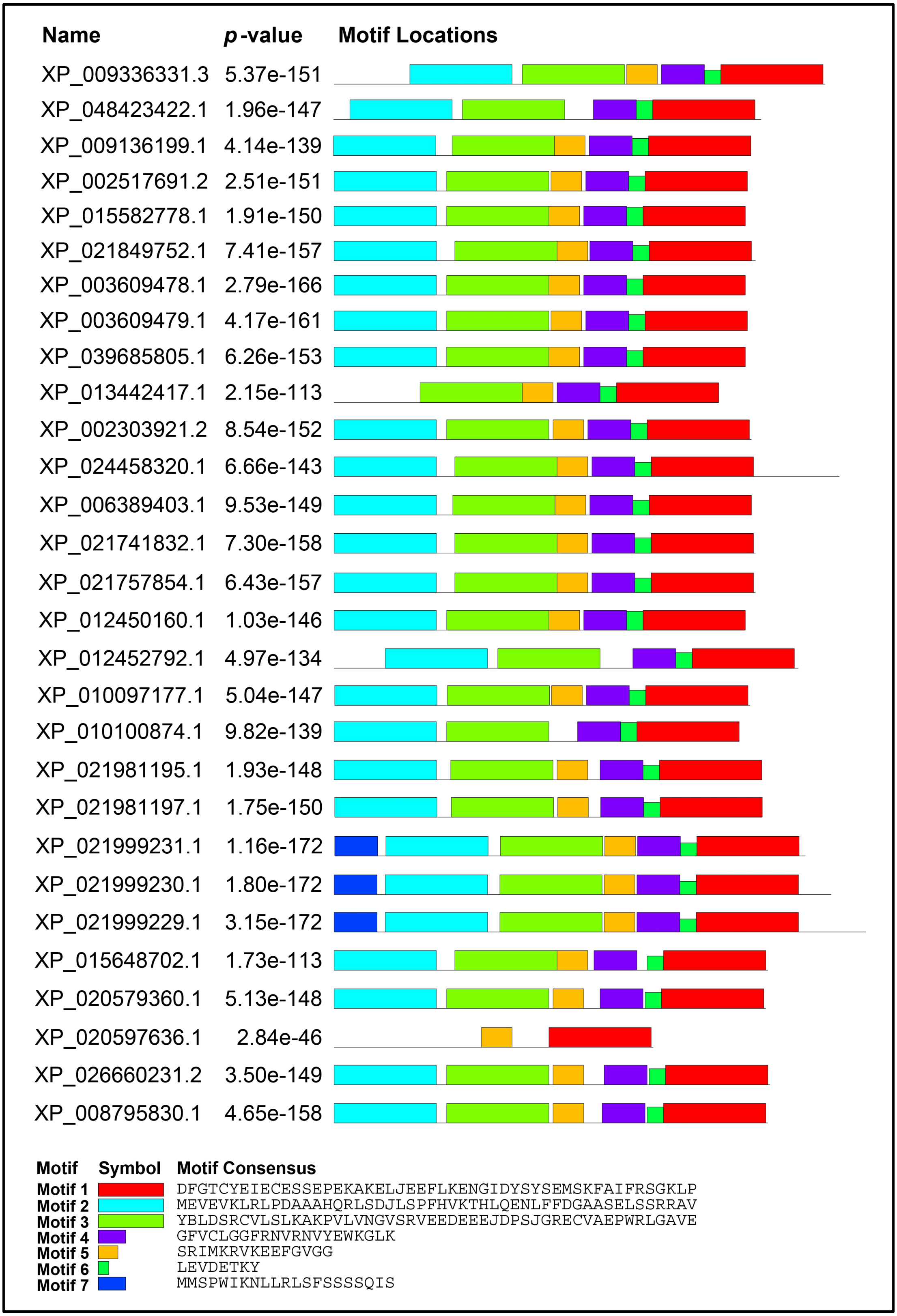

Supplementary Figure 1
Motif analysis of PbrTTMs and orthologous proteins. The motif analysis was conducted with MEME Suite (version 5.5.0).

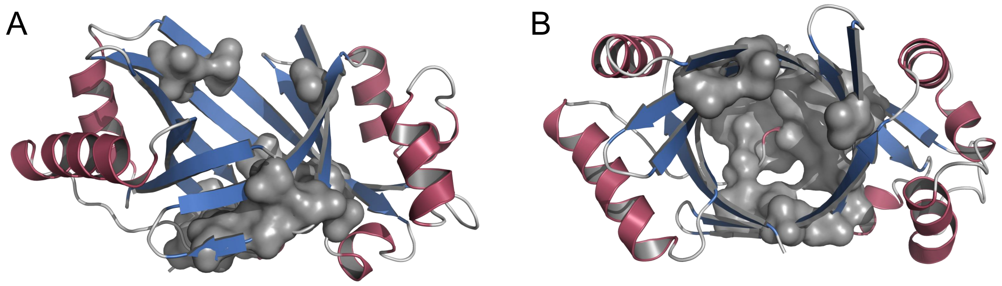

Supplementary Figure 2
Distribution of hydrophobic residues in the catalytic tunnel. Hydrophobic residues were presented with surface structure. From the vertical (A) and front (B) view of PbrTTM1, it could be observed that hydrophobic residues marked with gray cartoon mode mainly locate off the tunnel.
